# Elevated serum interleukin-10 level and M2 macrophage infiltration are associated with poor survival in angioimmunoblastic T-cell lymphoma

**DOI:** 10.18632/oncotarget.19301

**Published:** 2017-07-17

**Authors:** Jun Soo Ham, Ha Young Park, Kyung Ju Ryu, Young Hyeh Ko, Won Seog Kim, Seok Jin Kim

**Affiliations:** ^1^ Division of Hematology and Oncology, Department of Medicine, Samsung Medical Center, Sungkyunkwan University School of Medicine, Seoul, Korea; ^2^ Department of Pathology, Inje University Busan Paik Hospital, Busan, Korea; ^3^ Department of Health Sciences and Technology, Samsung Advanced Institute for Health Sciences and Technology, Sungkyunkwan University, Seoul, Korea; ^4^ Department of Pathology, Samsung Medical Center, Sungkyunkwan University School of Medicine, Seoul, Korea

**Keywords:** IL-10, M2 macrophage, angioimmunoblastic T-cell lymphoma

## Abstract

Interleukin-10 (IL-10) induces an immunosuppressive microenvironment including M2 macrophages, inhibiting anti-tumor immunity. The aim of this study was to evaluate whether serum IL-10 level at diagnosis and tissue infiltration of M2 macrophages could predict survival outcome of patients with angioimmunoblastic T-cell lymphoma (AITL).

We measured serum levels of IL-5, IL-10, IL-12, and interferon-gamma (IFN-γ) at diagnosis in AITL and other common subtypes of nodal T-cell lymphoma including peripheral T-cell lymphoma, not otherwise specified (PTCL-NOS), ALK-negative anaplastic large cell lymphoma (ALCL) or ALK-positive ALCL between September 2008 and December 2014. We also analyzed the infiltration of CD68- and CD163-positive macrophages in tumor tissue of AITL. In total, 97 patients with AITL (n=37), PTCL-NOS (n=40), ALK-negative ALCL (n=11), or ALK-positive ALCL (n=9) were treated with CHOP (cyclophosphamide, doxorubicin, vincristine, and prednisone). Among cytokines, only the serum level of IL-10 was significantly higher in AITL patients than in other subtypes (P < 0.05). Compared to other subtypes, the association of serum IL-10 with overall survival (OS) was only significant in AITL. Accordingly, the response to CHOP chemotherapy was significantly worse in the high IL-10 group, and infiltration of CD163-positive M2 macrophages was significantly associated with OS in AITL. In conclusion, this study demonstrated the prognostic relevance of serum IL-10 and tissue infiltration of M2 macrophages in AITL patients. Our results suggest the possible use of these variables as potential therapeutic targets and novel prognostic indicators in patients with AITL.

## INTRODUCTION

Angioimmunoblastic T-cell lymphoma (AITL) is the second most common subtype of peripheral T-cell lymphomas (PTCLs) according to the International T-Cell Lymphoma Project [1]. AITL was originally described as “angioimmunoblastic lymphadenopathy with dysproteinemia (AILD)” and is characterized by frequent relapses and poor prognosis [2]. Analysis of molecular profiles revealed that AITL is derived from follicular T-helper cells [3, 4]. The pathological characteristics of AITL such as infiltration of inflammatory cells and angiogenesis underline the importance of the microenvironment in this disease entity. Accordingly, the aggressiveness of AITL might be more influenced by cytokines and tumor microenvironment than other subtypes of PTCL such as peripheral T-cell lymphoma, not otherwise specified (PTCL-NOS) and anaplastic large cell lymphoma (ALCL). Thus, analysis of serum cytokines might help to understand the biological characteristics and identify patients at high risk of AITL. A recent study of gene expression profiles of PTCLs showed that expression of GATA-binding protein 3 (GATA3) could identify a high-risk subset of PTCL-NOS [5, 6]. GATA3 is a transcription factor related to type 2-helper (T_h_2) cells; high GATA3 expression can induce T_h_2-associated cytokines such as interleukin (IL)-4, IL-5, IL-10, and IL-13 [6]. As interleukins such as IL-4 and IL-10 can increase the level of M2 polarized macrophages, thus inhibiting the anti-tumor effect of non-neoplastic T-cells, T_h_2-associated cytokines can influence the tumor microenvironment to promote tumor progression [7]. Consistent with the effects of GATA3 and T_h_2-associated cytokines on the tumor microenvironment, a recent study also demonstrated the correlation of GATA3 expression with macrophage infiltration of the tumor, resulting in poor prognosis in patients with PTCL [8]. However, there are few studies on the role of serum GATA3-related cytokines in the promotion of type 2-helper cells, the so-called T_h_2-associated cytokines, and tumor-associated macrophages in patients with AITL. Therefore, we measured serum levels of T_h_2-associated cytokines at diagnosis in AITL and two other common subtypes of nodal PTCL (PTCL-NOS and ALCL). The association of T_h_2-associated cytokines with survival outcome in AITL was compared with that in PTCL-NOS and ALCL. Furthermore, we analyzed the prognostic value of M2 polarized macrophages in AITL to demonstrate the role of T_h_2-associated cytokines and tumor-associated macrophages in the aggressiveness of AITL.

## RESULTS

### Characteristics of patients

**Figure 1:**
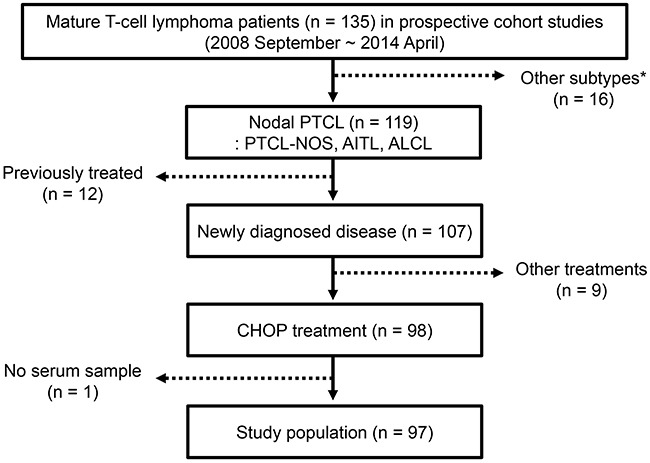
Consort diagram Patients with nodal PTCL were selected from 135 patients with mature T-cell lymphoma enrolled in prospective cohort studies from September 2008 to April 2014. The study population fulfilling the inclusion criteria for the study consisted of 97 patients with PTCL-NOS, AITL, or ALCL. PTCL = peripheral T-cell lymphoma; CHOP = cyclophosphamide, doxorubicin, vincristine, and prednisolone. *Other diagnosis: Cutaneous T-cell lymphoma, hepatosplenic T-cell lymphoma, enteropathy-associated T-cell lymphoma.

**Figure 2:**
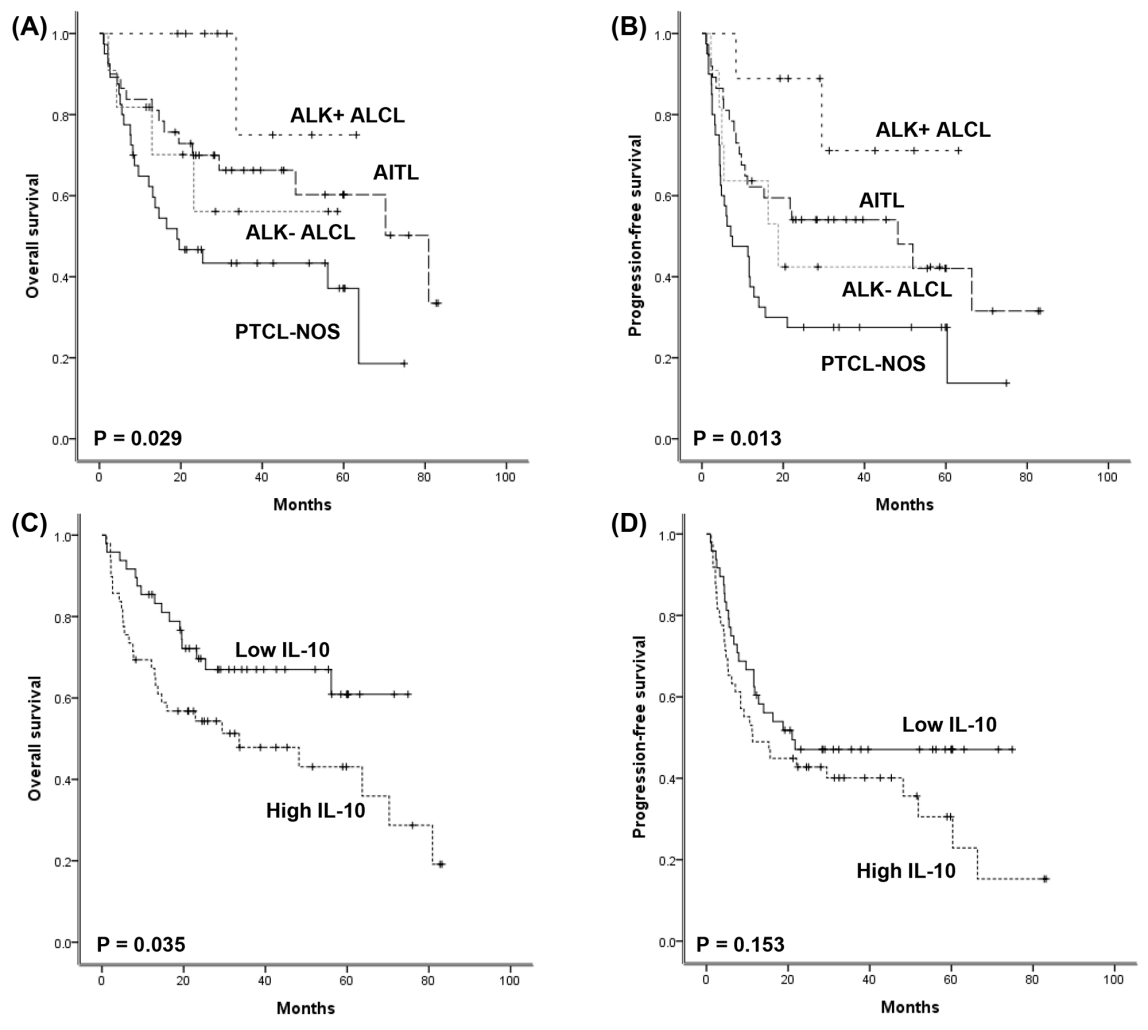
**(A, B)** Comparison of overall and progression-free survival among four subtypes. **(C, D)** Comparison of overall and progression-free survival between high and low serum IL-10 groups.

Based on the selection criteria, 97 patients were enrolled as the study population for this study out of 119 patients with AITL, PTCL-NOS, or ALCL (Figure 1). The median age at diagnosis was 59 years (range: 18–85 years), and the male to female ratio was 2:1 (Table 1). AITL and PTCL-NOS accounted for 79% of cases whereas ALK-negative and -positive ALCL were 21%. Thirteen patients (13%) had stage I/II, and 86 patients (87%) had stage III/IV disease. More than 60% of patients had elevated level of serum lactate dehydrogenase. Classification according to International Prognostic Index (IPI) risk showed that 58% of patients (n = 56) were high or high-intermediate risk. All patients received CHOP chemotherapy, including 13 patients receiving everolimus plus CHOP and seven patients receiving bortezomib plus CHOP. After diagnosis, 66 patients (68%) completed the planned number of CHOP chemotherapy (six) cycles, whereas the remaining 31 patients failed to receive all cycles of chemotherapy due to disease progression or treatment-related morbidity. The response was as follows: complete response (CR, n = 64), partial response (PR, n = 11), and progressive disease (PD, n = 22). At the time of analysis, 58 events including 44 deaths were documented, and the median PFS and OS were 16.3 months (95% CI: 6.5 - 26.1 months) and 63.7 months (95% CI: 32.1–95.3 months), respectively. The characteristics of patients at diagnosis were not significantly different among patients with AITL, PTCL-NOS and ALK-negative/positive ALCL (Table 2). Nevertheless, the PFS and OS were significantly different according to the subtype of PTCL (Figure 2A, 2B). Especially, the survival of AITL patients showed a superior outcome to that of PTCL-NOS patients.

**Table 1:** Characteristics of patients (n = 97)

Characteristics		n	%
**Age (years)**	≤ 60	55	57
	> 60	42	43
**Sex**	Male	65	67
	Female	32	33
**Histology**	PTCL-NOS	40	41
	AITL	37	38
	ALK-/ALK+ ALCL	11/9	21
**Performance status**	ECOG 0/1	71	73
	ECOG ≥2	26	27
**Serum LDH**	Normal	37	38
	Increased	60	62
**B symptoms**	Absent	50	52
	Present	47	48
**Number of extranodal involvements**	0/1	61	63
	≥ 2	36	37
**Ann Arbor stage**	I/II	5/8	13
	III/IV	31/53	87
**Bone marrow involvement**	No	63	65
	Yes	34	35
**International Prognostic Index**	Low/Low-intermediate	23/18	42
	High-intermediate/High	33/23	58

PTCL = peripheral T-cell lymphoma; ECOG = Eastern Cooperative Oncology Group; LDH = lactate dehydrogenase; AITL = angioimmunoblastic T-cell lymphoma; ALK = anaplastic lymphoma kinase; ALCL = anaplastic large cell lymphoma.

**Table 2:** Comparison of characteristics of patients according to subtypes

Characteristics		PTCL	AITL	ALK-/+ALCL	P
		n = 40	n = 37	n = 11/9	
**Age (years)**	≤ 60	24	17	7/7	0.288
	> 60	16	20	4/2	
**Sex**	Male	30	22	7/6	0.539
	Female	10	15	4/3	
**Performance status**	ECOG 0/1	29	26	10/6	0.547
	ECOG ≥2	11	11	1/3	
**Serum LDH**	Normal	13	14	5/5	0.583
	Increased	27	23	6/4	
**B symptoms**	Absent	24	15	7/4	0.288
	Present	16	22	4/5	
**Extranodal involvements**	0/1	22	25	8/6	0.588
	≥ 2	18	12	3/3	
**Ann Arbor stage**	I/II	5	5	1/2	0.848
	III/IV	35	32	10/7	
**Bone marrow involvement**	No	24	23	9/7	0.461
	Yes	16	14	2/2	
**IPI**	L/LI	8	9	3/3	0.837
	HI/H	32	28	8/6	

PTCL = peripheral T-cell lymphoma; AITL = angioimmunoblastic T-cell lymphoma; ALK = anaplastic lymphoma kinase; ALCL = anaplastic large cell lymphoma; LDH = lactate dehydrogenase; ECOG = Eastern Cooperative Oncology Group; IPI = International Prognostic Index; L = low; LI = low-intermediate; HI = high-intermediate; H = high.

### Measurement of serum cytokines

Out of seven cytokines evaluated, IL-2, IL-4, and IL-13 were each detected in fewer than 10 patients (n = 2, 6, and 3, respectively) and were therefore excluded from the analysis. IL-10, IL-12, and IFN-γ were measured in all patients, and the median value and range of serum levels of these three cytokines were as follows: IL-10 (1.91 pg/mL, range: 0.34– 7626.5 pg/mL), IL-12 (1.07 pg/mL, range: 0.62–27.8 pg/mL), and IFN-γ (6.67 pg/mL, range: 4.17–87.4 pg/mL), respectively. IL-5 was detected in 63 patients at a median level of 0.48 pg/mL (range: 0.02–22.2 pg/mL). The median serum level of IL-10 was significantly higher in AITL (3.98 pg/mL) than in PTCL-NOS (1.41 pg/mL) and ALCL (1.08 pg/mL, P = 0.016). Serum levels of other cytokines were not significantly different among AITL, PTCL-NOS, and ALK-negative/positive ALCL (P > 0.05). Clinical and laboratory characteristics including stage, serum LDH, number of extranodal involvements, and IPI risk were not significantly associated with serum levels of these cytokines (P > 0.05, data not shown).

### Association of serum cytokines with survival outcome

**Figure 3:**
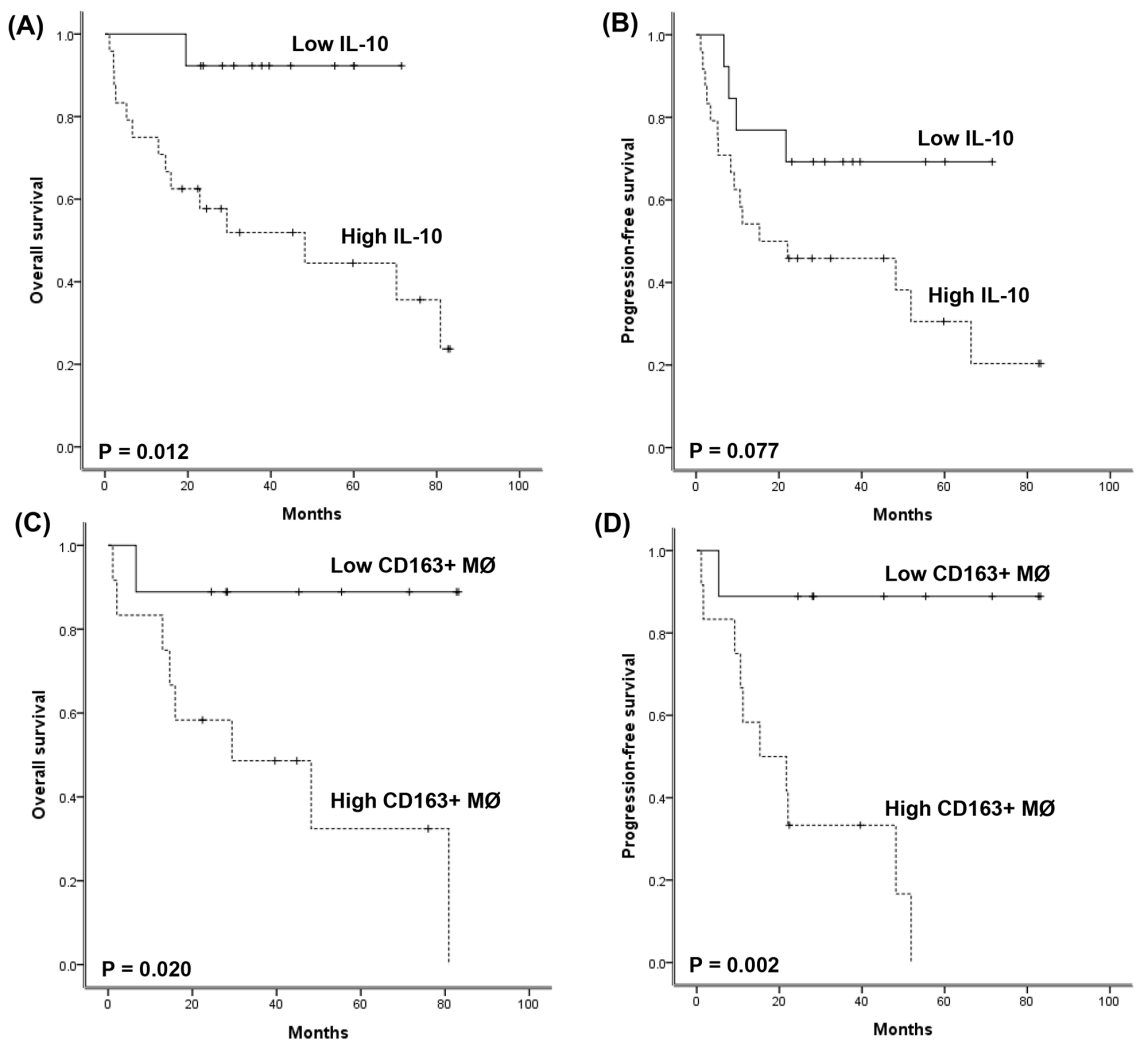
**(A, B)** Comparison of overall and progression-free survival between high and low serum IL-10 groups in AITL patients. **(C, D)** Comparison of overall and progression-free survival between high and low CD163-positive macrophage groups among AITL patients.

The cutoff values of IL-10, IL-12, and IFN-γ were determined as 1.845, 1.060, and 6.645 pg/mL, respectively according to the ROC curves. Thus, the high- and low-groups were defined as greater than or equal to the cutoff and less than the cutoff value, respectively. When patients were categorized into high (n = 49) and low (n = 48) IL-10 groups, the high IL-10 group showed inferior OS compared with the low IL-10 group (Figure 2C, P = 0.035), although IL-10 level failed to show a significant association with PFS (Figure 2D, P = 0.153). High IL-12 and high IFN-γ groups were not significantly associated with OS and PFS (P > 0.05, data not shown). Similarly, dichotomization of patients based on the presence of IL-5 was not associated with survival outcomes (P > 0.05, data not shown). Consistent with the association of serum IL-10 with OS, the response to CHOP chemotherapy was significantly associated with IL-10 level; the low IL-10 group showed a higher CR rate (38/48, 79% vs. 26/49, 53%) and lower PD rate (6/48, 13% vs. 16/49, 33%) than the high IL-10 group. However, this association between IL-10 and response to CHOP chemotherapy was only significant in AITL in the subgroup analysis according to subtype. Thus, among 37 patients with AITL, 31 patients achieved CR to CHOP, but six cases with disease progression was only found in the high IL-10 group (P = 0.049). Accordingly, there was a significant association of serum IL-10 with OS in AITL (Figure 3A, P = 0.012). The high IL-10 group also showed a trend of inferior PFS compared with the low IL-10 group in AITL (Figure 3B, P = 0.077). However, this relationship between IL-10 and survival outcome was not demonstrated in the subtypes PTCL-NOS and ALCL (P > 0.05, Supplementary Figure 1).

### Tumor-associated macrophage infiltration in AITL

**Figure 4:**
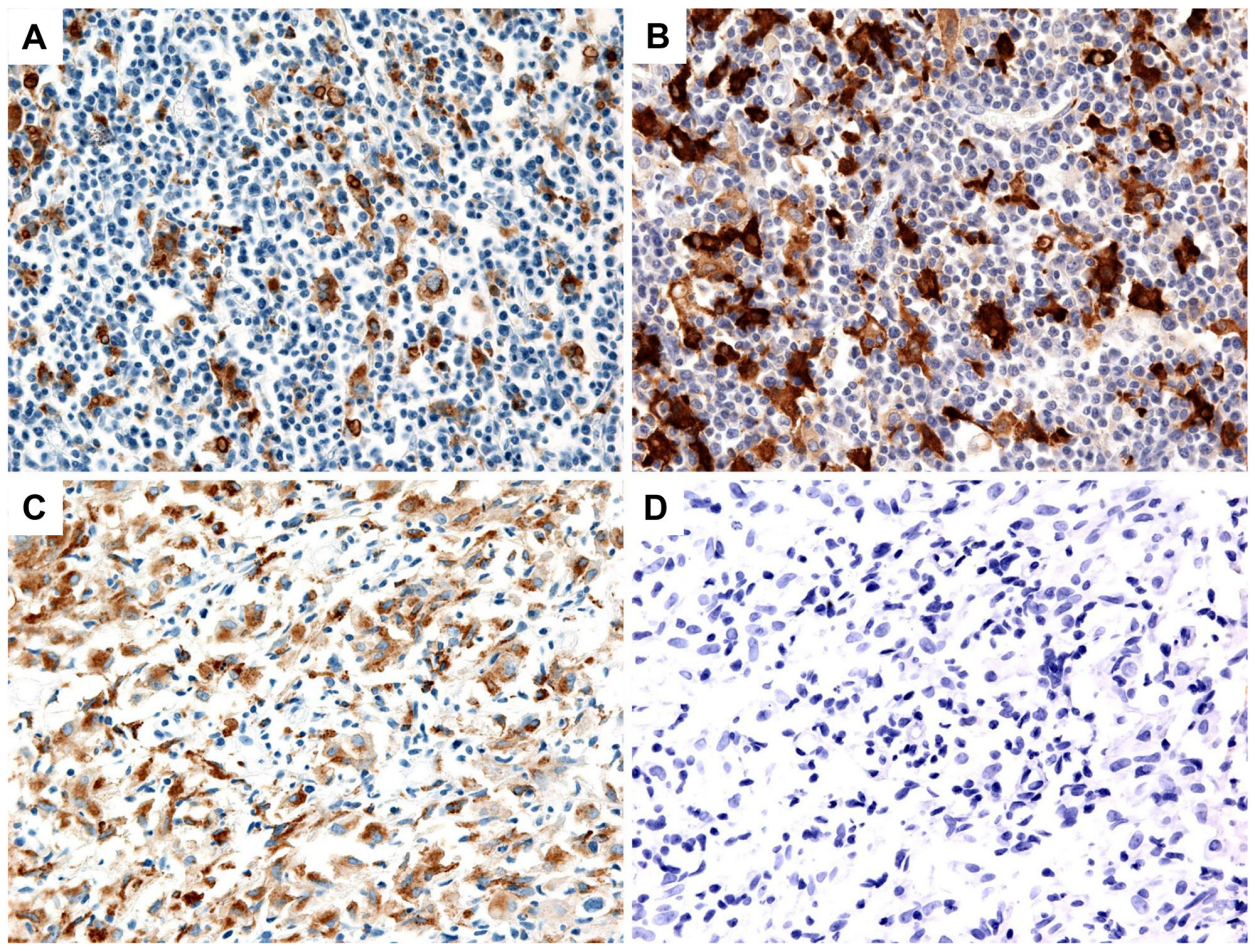
Representative images of high and low infiltration of CD68-positive and CD163-positive macrophages in tumor tissue **(A, B)** High infiltration of CD68-positive and CD163-positive macrophages. **(C)** Low infiltration of CD68-positive macrophages. **(D)** Absence of CD163-positive macrophages.

Given the significant association of serum IL-10 with overall survival in patients with AITL, we analyzed the infiltration of tumor-associated macrophages in tumor tissue of 21 AITL patients who had archived paraffin-embedded tissue blocks available for immunohistochemical staining. Infiltration of CD68-positive macrophages and CD163-positive M2 macrophages was found in tumor tissue of AITL (Figure 4A-4D). The median percentage of CD68-positive and CD163-positive macrophages was 8.5% (range: 1.3–22.5%) and 2.8% (0.3–32.3%), respectively. Infiltration of CD163-positive macrophages was significantly correlated with infiltration of CD68-positive macrophages (*r* = 0.666, P = 0.001). However, when patients were dichotomized into high and low groups according to the median percentage of CD68- and CD163-positive macrophages, infiltration of CD68-positive macrophages was not related with survival outcomes (P > 0.05), whereas infiltration of CD163-positive M2 macrophages was significantly associated with both OS and PFS (P < 0.05, Figure 3C, 3D). The mean level (± standard deviation) of serum IL-10 in the high group of CD163-positive macrophages (n = 12) was 58.84 pg/mL (± 175.29pg/mL), whereas that of the low group of CD163-positive macrophages (n = 9) was 9.80 pg/mL (± 16.77pg/mL) although this difference was not statistically significant due to the relatively small number of patients (n = 21, P > 0.05).

## DISCUSSION

Our study evaluated the serum levels of cytokines, especially T_h_2-associated cytokines, and analyzed their association with survival outcome in patients with nodal T-cell lymphomas including AITL, PTCL-NOS, and ALK+/- ALCL. Although other interleukins failed to show a significant association with survival, elevated serum level of IL-10 was significantly associated with a poor response to CHOP chemotherapy as well as inferior overall survival (Figure 2C). However, subgroup analysis showed that this association of serum IL-10 with survival was only significant in AITL patients (Figure 3A). Thus, our findings implied that IL-10 might contribute to tumor aggressiveness in AITL rather than other subtypes. IL-10 is one of the T_h_2-associated cytokines and induces an immunosuppressive microenvironment such as infiltration of tumor-associated macrophages, thus inhibiting the anti-tumor immune reaction. As high expression of IL-10 is associated with the accumulation of tumor-associated macrophages in follicular and large B-cell lymphoma [9, 10], the association of elevated serum IL-10 level with inferior survival of AITL could be explained by the IL-10–dependent accumulation of tumor-associated macrophages. We therefore evaluated the prognostic value of tumor-associated macrophages in AITL because our data showed a significant association of high serum IL-10 with poor OS in AITL. Although the number of patients was relatively small, the extent of CD163-positve macrophage infiltration was significantly associated with poor OS and PFS (Figure 3C, 3D). This result was consistent with a previous study reporting the association of M2 macrophages with inferior survival of AITL rather than CD68-positive macrophages, because CD163 is a marker of M2 macrophages [11]. As M2 macrophages express PD-L1, the binding of M2 macrophages to cytotoxic T-cells through the interaction of PD-1 and PD-L1 could inhibit T-cell function [7]. Thus, the accumulation of M2 macrophages could contribute to the development of an immunosuppressive tumor microenvironment that promotes tumor cell growth, and targeting IL-10–induced M2 macrophage polarization might be a potential therapeutic approach for AITL. However, the clinical application of tumor-associated macrophages in the management of AITL has some obstacles. First, the quantification of macrophage infiltration might be difficult to perform in a reproducible way. Second, the extent of infiltration as well as the pattern of distribution within tumor mass also might be important. Thus, more studies are needed to solve these problems in the future.

In clinical practice, the treatment strategy for AITL is mainly based on anthracycline-containing chemotherapy such as CHOP (cyclophosphamide, doxorubicin, vincristine, and prednisolone) or CHOP-like regimens, as for other nodal PTCLs [12, 13]. However, the treatment outcome is so unsatisfactory that a clinical trial is still preferred for newly diagnosed AITL. As a result, various new agents have been combined with CHOP, including anti-CD52 monoclonal antibody (alemtuzumab), proteasome inhibitor (bortezomib), anti-vascular endothelial growth factor antibody (bevacizumab), denileukin diftitox, and mTOR inhibitor (everolimus), in order to augment the efficacy of CHOP chemotherapy in newly diagnosed PTCL patients [14–18]. However, none of these combinations was promising because the duration of response was relatively short and many patients eventually suffered relapse. If blockade of IL-10–mediated M2 macrophage polarization could be combined with CHOP chemotherapy or immune checkpoint inhibitors such as pembrolizumab, the treatment outcome of AITL might be improved. As IL-10–dependent M2 macrophage polarization is mediated through JAK-STAT signaling, and inhibition of M2 macrophage polarization was demonstrated by an *in vitro* study showing that ruxolitinib inhibited T_h_2-associated cytokines-induced M2 macrophage polarization [6], the use of ruxolitinib might have potential as a new treatment for AITL. Because tumor cells of AITL are derived from follicular helper T-cell, the IL-10–induced M2 macrophage polarization also might contribute to the outcome of PTCL-NOS with follicular helper T-cell variant. However, our patients with PTCL-NOS did not include the case with follicular helper T-cell variant. Thus, further study with large study population should be warranted to validate our findings in follicular helper-T-cell derived T-cell lymphomas including AITL.

In conclusion, our study demonstrated the prognostic relevance of serum IL-10 and tissue infiltration of M2 macrophages in AITL, suggesting their possible application as potential therapeutic targets and new prognostic indicators in patients with AITL.

## MATERIALS AND METHODS

### Study design

We performed this study to evaluate the prognostic value of serum T_h_2-associated cytokines at diagnosis in newly diagnosed AITL patients. The primary objective was to analyze the association between serum levels of T_h_2-associated cytokines and survival outcomes of AITL and other nodal T-cell lymphomas. The secondary objective was to evaluate their relationship with clinical characteristics and macrophage infiltration in tumor tissue of AITL. We selected study patients from non-Hodgkin lymphoma patients enrolled in our prospective cohort studies between September 2008 and December 2014 according to the following criteria. First, patients should be newly diagnosed with AITL, PTCL-NOS, or ALCL according to the pathology criteria of the World Health Organization [19]. Second, patients should receive CHOP as a primary treatment after diagnosis. Third, patients should have serum samples available for measurement of T_h_2-associated cytokines. In our cohort studies, serum samples were stored at −80°C until analysis after receiving written informed consent. The Institutional Review Board of our institute approved all aspects of those cohort studies, and two studies were registered at www.clinicaltrials.gov (first study: NCT#00822731; second study: NCT#01877109). For the analysis of survival outcome, we updated the survival status at the time of analysis in June 2016.

### Patients

The clinical data of patients including pathological subtype, age, performance status, Ann Arbor stage, serum lactate dehydrogenase (LDH), number of extranodal involvements, bone marrow involvement, B symptoms, treatment, and clinical outcomes were recorded during the prospective cohort studies. All patients were planned to receive six cycles of CHOP therapy after diagnosis. CHOP chemotherapy consisted of intravenous administration of cyclophosphamide (750 mg/m^2^), doxorubicin (50 mg/m^2^), and vincristine (1.4 mg/m^2^) on day 1 and oral administration of prednisolone (100 mg daily) on days 1-5. A group of patients enrolled in a phase II study received bortezomib or everolimus in combination with CHOP as previously reported [15, 18]. The response evaluation was performed after the 3^rd^ and 6^th^ cycles of chemotherapy, and the response was determined by CT and PET/CT scan according to the revised response criteria for malignant lymphoma [20].

### Multiplex cytokine assay

Serum samples were collected at diagnosis and stored at -80°C until analysis. Serum aliquots had not been previously thawed before use in the multiplex chemokine assay. We measured the levels of IL-2, IL-4, IL-5, IL-10, IL-12, IL-13, and interferon-gamma (IFN-γ) in triplicate with the Procarta cytokine profiling kit (Panomics, CA, USA) using the Bio-Plex Cytokine Assay System (Bio-Rad Laboratories, Hercules, CA, USA) according to the manufacturer's instructions.

### Immunohistochemistry

Whole-section samples fixed in 10% neutral buffered formalin and embedded in paraffin wax were used for immunostaining. Serial sections were used for staining with antibodies against two common macrophage antigens: one for staining with anti-CD68 (Dako, Glostrup, Denmark) and one for staining with anti-CD163 (Novocastra, Newcastle, UK). Staining was performed using the BOND-MAX autostaining system (Leica Biosystems, Newcastle, UK) according to the manufacturer's protocol. To count the numbers of CD68-positive and CD163-positive macrophages, we evaluated three different high-power fields (an area of 0.196 mm^2^/high-power field for a total area of 0.588 mm^2^) for each stain.

### Statistics

The optimal cutoff value for each cytokine was determined by receiver-operating characteristic (ROC) curve analysis. ROC curve analysis was performed using MedCalc (Version 12.7, Medcalc Software, Ostend, Belgium). The association of cytokine production with clinical and laboratory parameters was analyzed by the Chi square test. The coefficient of correlation (*r*) between the expression levels of markers was calculated using the Spearman rank-sum test. The comparison of mean values was done by the Mann-Whitney *U* test. The Kaplan–Meier method was used for univariate analysis of survival outcomes, and survival outcomes were compared by the log-rank test. Progression-free survival (PFS) was defined as the time from the date of diagnosis to the date of documented disease progression or any kind of death, whereas overall survival (OS) was measured from the date of diagnosis to the date of death from any cause and was censored at the date of the last follow-up visit. All statistical analyses were conducted using the IBM PASW software package (version 22.0; SPSS Inc., Chicago, IL, USA).

## SUPPLEMENTARY MATERIALS FIGURE


