# Supplementary material for: Elevated serum interleukin-10 level and M2 macrophage infiltration are associated with poor survival in angioimmunoblastic T-cell lymphoma
Source: Oncotarget. 2017 Jul 17;8(44):76231–40. doi: 10.18632/oncotarget.19301 (PMC5652701; doi:10.18632/oncotarget.19301)
Supplement: Supplementary file 1 [file oncotarget-08-76231-s001.pdf]

Elevated serum interleukin-10 level and M2 macrophage infiltration are associated with poor survival in angioimmunoblastic T-cell lymphoma

SUPPLEMENTARY MATERIALS

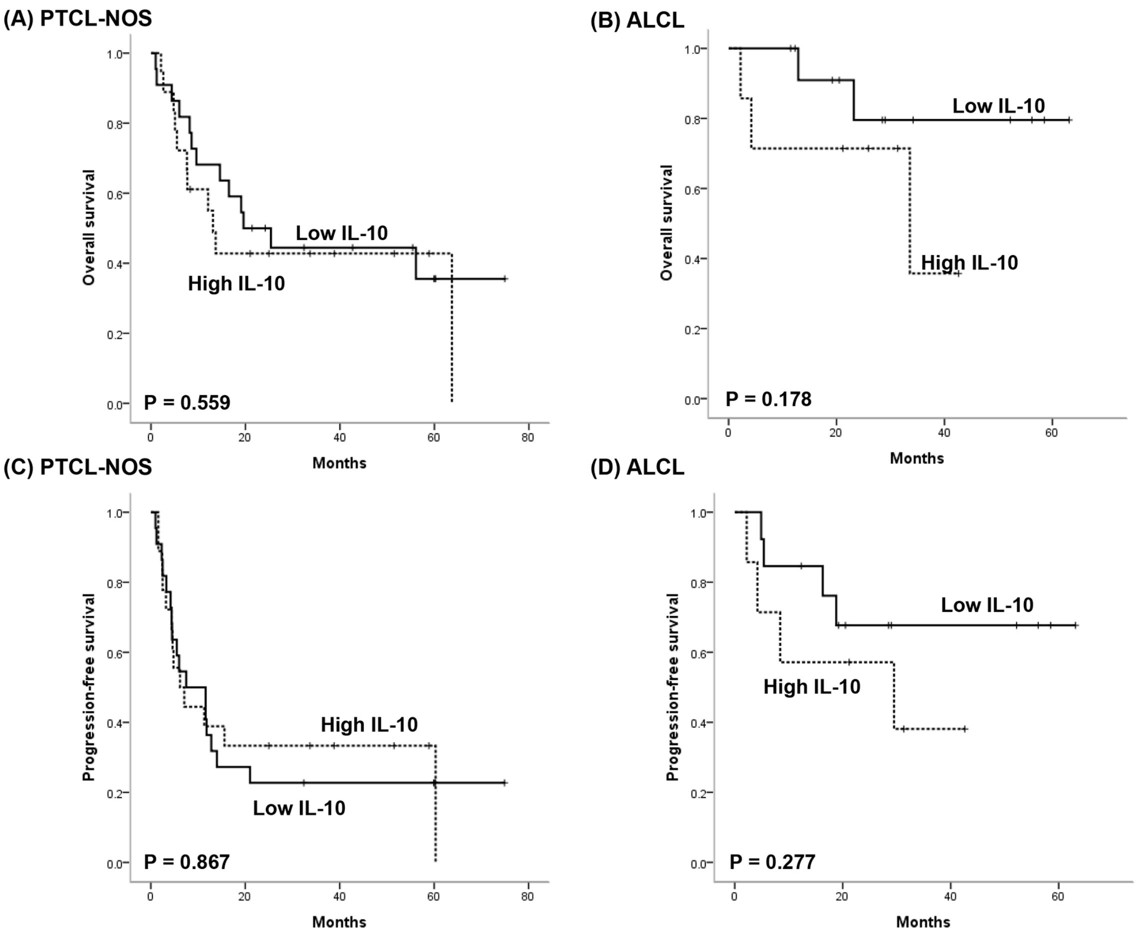

Supplementary Figure 1: Comparison of OS and PFS according to high and low serum IL-10 groups.
